# The Efficiency of Cervical Pap and Comparison of Conventional Pap Smear and Liquid-Based Cytology: A Review

**DOI:** 10.7759/cureus.48343

**Published:** 2023-11-06

**Authors:** Shirin Dasgupta

**Affiliations:** 1 Dr. B. C. Roy Multi Speciality Medical Research Centre, Indian Institute of Technology Kharagpur, Kharagpur, IND

**Keywords:** liquid-based cytology, diagnostic efficacy, conventional pap smear, cervical intraepithelial neoplasia (cin), cervical cancer

## Abstract

Cervical cancer is one of the leading health burdens globally with a huge incidence in developing countries like India. Cervical cancer has an extended premalignant stage known as cervical dysplasia or cervical intraepithelial neoplasia (CIN). CIN can be grade 1, 2, or 3 depending on its severity. One of the most effective methods of cervical cancer screening and prevention is detecting these premalignant lesions by cervical cytology.

Pioneered by Dr. George Nicholas Papanicolaou, the Papanicolaou (Pap) stain became an important advent for the microscopic evaluation of the exfoliated cervical cells. Over the years, this method of conventional Pap smear became more practiced and yielded excellent results, so much so that the incidence of cervical cancer actually started to decline in developed countries. However, few drawbacks started to become evident with conventional Pap smears like unsatisfactory samples due to obscuring materials, false negative results due to sampling error, and low sensitivity.

To overcome these drawbacks of conventional Pap smear, liquid-based cytology (LBC) was introduced in 1996. Thereafter, many investigations and studies have been conducted by many authors to compare the efficacy of conventional Pap smear and LBC. This review puts forward the facts and results of various studies pertaining to efficacy of cervical cytology, comparing conventional Pap smear and LBC, and highlighting the pros and cons of each method based on various studies. For this review, relevant articles under the headings “Conventional PAP smear”, “Cervical cancer screening”, “Liquid-based cytology” and “Comparison” have been searched in PubMed/MEDLINE and Google Scholar. About 100 articles were studied, and all the facts have been highlighted.

While many studies did support LBC over conventional Pap smear for screening of cervical abnormalities, some studies did not find any major difference between the two and preferred the practice of conventional Pap smear in our Indian scenario considering the low-resource setting and low price.

This research highlights the various facts of the two types of cervical Pap smear and their comparison.

## Introduction and background

Cervical cancer is a rising health issue that is a leading cause of woman mortality globally and its incidence is upsurging. The difference in the incidence between developed countries wherein cervical cancer has markedly reduced and developing countries is huge. Developing countries like India carry a huge burden of cervical cancer. World Cancer statistics point out that >80% of all cervical cancer cases are found in developing and low‑resource countries, the reason being a lack of awareness and struggle in effective execution of cervical screening programs [1]. India harbors more than one-fifth of all deaths due to cervical cancer [2].

Cervical cancer is a preventable disease due to the long premalignant stage [3]. Implementation of a vigorous screening program can help in early detection and timely management [4]. Cervical dysplasia (a preinvasive form of cervical cancer) is an important health hazard affecting women of all ages [5,6].

In 1947, the concept of cervical preinvasive state was familiarized, when it was established that epithelial changes having the appearance of invasive cancer were recognized but these changes were confined to the epithelium. These lesions if left untreated can evolve to cervical cancer. Such early precursor lesions are known as cervical dysplasia. In 1968, the term cervical intraepithelial neoplasia (CIN) was coined. This term is synonymous with dysplasia which means abnormal maturation [7]. Detecting and removing cervical dysplastic lesions before they progress into invasive cancer is the cornerstone of cervical cancer screening programs.

Papanicolaou (Pap) smear is one of the effective methods of screening for cervical abnormalities. The Pap smear test is a mode of cervical screening that can be utilized to identify potentially precancerous and cancerous lesions of the cervix. The typical method for a Pap smear collection is to sample the junction between the ectocervical and endocervical canal (transformation zone), typically via a wooden spatula and an endocervical brush, then smear these samples onto a glass slide and immediately fix the smear with a fixative [7]. The reporting is done as per The Bethesda System for Reporting Cervical Cytology. Due to widespread cervical cytology screening programs, there has been a significant reduction in mortality from cervical cancer in developed countries [8]. The commencement of a cervical cancer screening program comprising cervical cytology as well as human papillomavirus testing witnessed a significant decline in cervical cancer deaths in developed countries [9].

Two techniques are followed for cervical cytology. One is the conventional Pap smear, which to date is the most frequently used technique for cervical cytology, because of its easy availability and cheapness. The other technique is liquid-based cytology (LBC). Even though, it is the most acquainted technique of cervical cancer screening in developed countries, its’ availability is limited to a few centers in developing countries the reason being it is costly and requires high-end infrastructure [10].

Even though it is easy to conduct, conventional Pap smears have a few technical shortcomings. The bulk of the collected cellular material is wasted due to improper collection of samples from the patient onto the sampling device and due to inadequate transfer of cells from the sampling device (brush) into the glass slide. Furthermore, suspicious cells microscopically present on the slide are often missed because of inflammatory or hemorrhagic background, causing underdiagnosis. In contrast to conventional Pap smears, LBC provides a monolayered thin smear with a clearer background. Additionally, in the case of LBC, the collected material can be conserved for further studies, if required [10]

The aim of consultants is to collect the sample and minimize the sampling error via effective sampling of the cervical transformation zone as well as dismissal of drying artifacts via rapid fixation. Regardless, false-negative results take place with the conventional Pap technique in at least 20% of the instances, the most common cause being errors in sampling techniques [11].

This high frequency of false negatives has paved the path to the formation of new diagnostic techniques [12]. Of late, LBC techniques like ThinPrep and SurePath have gained popularity, because evidence suggests their use has resulted in a reduction of the frequency of inadequate smears. LBC involves a similar method of sampling the cervix as is done in the conventional Pap smear, but after sampling the device is positioned in a liquid medium which is transported to the laboratory, and the cells are either collected via extraction across a filter in case of ThinPrep or by layering onto a density reagent in case of SurePath following which, the cells are placed evenly on a slide as a monolayer and reviewed. The benefits of these liquid-based methods include decreased obscuring materials and hemorrhage on the slide, a decrease in cellular misrepresentation, and an even cell distribution on the slide [11].

In this review article, we are going to review the utility and efficiency of cervical Pap smears in screening cervical abnormalities as well the compare the diagnostic utilities of the two types of cervical cytology as put forward by various studies.

## Review

Methodology

For this review, relevant articles under the headings “Conventional PAP smear”, “Cervical cancer screening”, “Liquid based cytology” and “Comparison” have been searched in PubMed/MEDLINE and Google Scholar. The author has gone through about 100 publications including original research and review articles pertaining to this topic. The inclusion criteria are those papers that have compared the two types of cervical Pap smear. The exclusion criteria are those papers that have only used either of the two methods for their study.

Pap smear in cervical cancer screening

Cervical cancer is the fourth most common disorder in women globally and the second most common cause of death due to cancer among Indian women [13]. This is an essentially preventable cancer, which can be possible through screening and intervention. The cervical cancer screening procedures use this slow-growing nature of the cancer as a tool for effective prevention.

The primary aetiology of cervical dysplasia and cancer is human papillomavirus (HPV) infection. But only an HPV infection is not enough to cause a cervical neoplasia which appears to be multifactorial and is associated with multiple etiological associations and risk factors. The other key risk factors reported are lack of education, poor socio-economic status, and disorders in sexual and reproductive behaviour. Therefore, evidence proposes that cervical cancer is open for primary and secondary prevention. In primary prevention, maintenance of sexual hygiene as well as the use of barrier contraceptives can indisputably result in the prevention of a considerable proportion of cervical cancers. However, primary prevention is largely governed by the general awareness among women about risk factors and lifestyle changes. General awareness is a foremost issue in developing countries where the educational level of women is yet to progress significantly. Hence, the main attention is on secondary prevention which can be implemented by early detection, according to the National Cancer Control Programme revised in 1985 [14].

Cervical cytology is one of the first-line methods of effective cervical cancer screening, which, by virtue of early identification and management of preinvasive lesions, has led to a steady drop in the incidence and death due to invasive cervical cancer. It is possible to detect early cervical anomalies (CIN) ages before invasive cancer sets in. This is the central idea behind cervical cytology screening [15]. Population-based screening with Pap smears has been used since the early sixties to detect cervical precancerous lesions. The epidemiological data show that well-organized cervical cancer screening with Pap smear had a significant impact on both mortality and morbidity from cervical cancer [16].

The history of cervical Pap smears goes way back. In 1928, it was first reported by Dr. George Nicolas Papanicolaou that malignant and abnormal cells of the cervix can be recognised in vaginal smears. He, along with gynaecologist, Herbet Traut delivered a thorough description of the female genital tract cytology along with the basis of the detection of unknown occult cancer in asymptomatic patients. Even though, at the outset, their observations were acknowledged with doubt both by clinicians and pathologists, later on, many of them confirmed their findings, thus identifying cervical smear as a routine screening test for identifying premalignant cervical lesions, known as “Pap test” or “Pap smear” [17].

In the 1940s, the first cervical cancer screening clinics were set up, where a number of women were screened for identification of early-stage uterine cancer. In one year, it was found that amongst 54 cases of cancer in 639 patients, 51 were correctly diagnosed by cytology and 6 were identified exclusively by Pap smear [18]. The employment of this very effective and simple screening test was trailed by an intense decline in the mortality rate due to cervical cancer in various populations. The use of Pap smears in the various screening programs of cervical cancer was given recognition in 1957 by the American Cancer Society [19].

Cervical cytology smears are reported microscopically by the Bethesda system, which was presented in 1988. The Bethesda system of reporting comprises uniform, clear, and reproducible terminology, which highlights the most recent facts of cervical neoplasia. It was then revised in 1999, 2001 and the latest in 2014. This reporting system includes an evaluation of specimen adequacy, if there is evidence of cervical abnormalities and if present, the severity of those abnormalities [20].

Cervical cytology is recommended as the primary screening tool for cervical cancers according to Current National Health and Medical Research Council guidelines, HPV testing being mostly limited to verification of cure after management of high‐grade lesions. The frequency of cervical cancer has significantly declined since the initiation of cervical cytology screening programs. It is understood that regular Pap screening tests can reduce cervical cancer diagnosis by up to 96% [21]. Conventional cervical Pap smear screening remains the primary method of cervical cancer detection [22,23].

A cervical cancer screening program in a population-based manner was carried out in Canada in 1949 [24]. This program was awarded with a reduction rate of 85% in the incidence of cervical cancer and mortality between 1955 and 1988, thus having a dramatic response [24]. Rigorous widespread cytology screening programs not only diminish the incidence and mortality due to invasive cervical carcinoma but also reduce the incidence of in situ and other precursor lesions of cancer cervix [25]. As cervical cancer screening programs gained more widespread recognition in the 1950s, the efficacy of conventional Pap smear was further explored by various population studies and it was established as the backbone of fruitful cytological cervical screening programs during that time [26].

LBC employs collecting the cervical smears and putting them in a liquid media following which they are processed in the laboratory, monolayered slides are prepared and reported. There are mainly two types of LBC, “ThinPrep” and “SurePap” which will be explained later.

Conventional Pap smear versus liquid-based cytology

A conventional Pap smear was the initial method of cervical cell examination wherein the cells exfoliated from the cervix was collected and viewed for any abnormalities. To date, many institutions and health setups follow conventional Pap smear testing for cervical cytology. Here, after positioning the patient in the lithotomy position, a Cusco’s speculum is used to locate the transformation zone (junction of endocervix and ectocervix). After fixing the speculum, an endocervical brush and an Ayer’s spatula are used to collect the sample from the transformation zone. Thereafter, the sample is smeared on a glass slide and fixed in ethyl alcohol for some time, stained with Pap stain and viewed under a microscope. Thus, the cervical sample taken with a cervical brush is directly transferred to the microscope slide for evaluation [27].

Many investigations have determined the conventional Pap smear test’s specificity to be about 98%-99%. However, its sensitivity ranges from 50% to 75% or less [28,29]. Furthermore, this method has an inadequacy rate extending from 5% to 25% [27]. The conventional Pap test has a number of shortcomings, which comprises inadequate cell transfer from the cytobrush to the slide, uneven distribution of cells, and the presence of obscuring materials like inflammatory cells, blood, and overlapped epithelial cells [30].

In May 1996, the Food and Drug Administration did provide recognition to LBC as an alternative to the conventional Pap smear with an aim to overcome the drawbacks [31]. In the last couple of decades, SurePath and ThinPrep (both LBC techniques) have substituted conventional Pap smear as the primary test method in a good number of cervical cancer screening programs [32]. In the ThinPrep method, a cervical brush is used to collect the cervical specimen after which the brush is rinsed in a vial containing a methanol-based preservative fluid. The brush is pushed to the bottom, bristles forced apart, brush swirled in the fluid thus releasing the cells. Later, the brush is thrown away. In the laboratory, vacuum filtration is used to isolate the cells from the fluid and subsequently transferred to the glass slide with the help of air pressure for adherence [32]. With SurePath, a broom-like device with a detachable head is used to collect the cervical sample. Post-collection, the head is detached and put in a vial with an ethanol-based preservative fluid. In the laboratory, centrifugation of the fluid is done for isolation of the cells from the fluid [32].

This processing results in a monolayer preparation that may be analysed by a computer-based imager and reviewed by a cytotechnologist.

During the past many years, different comparison studies have been conducted to compare conventional smears with LBC.

In spite of the best efforts in preparation of the conventional Pap smears, effectively only around 20% of cells are transferred to the slide besides having obscuring materials [33]. On the other hand, the LBC provides a clearer background with minimised cellular overlapping and the analyses can be more prompt compared to conventional smear [34].

Ronco et al. carried out a randomised controlled trial for comparison of the accuracy of conventional Pap smear with LBC for primary screening of cervical cancer [35]. This study concluded that there is no statistically significant difference in sensitivity between conventional Pap smear and LBC for detection of CIN of grade 2 or higher. However, LBC showed a significant decrease in unsatisfactory smears and had a lower positive predictive value than conventional Pap smears. There was also an increase in the incidence of low-grade lesions with LBC.

Monsonego et al. conducted a split-sample study in Europe which according to the authors, was the first multi-laboratory, large-scale assessment of the ThinPrep LBC [36]. This study concluded that not only the ThinPrep Pap Test is more precise than the conventional Pap smear but it is also a prospective technique to optimize the efficiency of cervical cancer screening.

Hashmi et al. conducted a study to compare the diagnostic utility of LBC and conventional Pap for detecting cervical epithelial lesions and found that LBC could detect squamous epithelial lesions at a rate higher than conventional Pap smear [10].

Another comparison study between LBC and conventional Pap smears stated unsatisfactory smears to be 7.1% with conventional Pap smears when compared to 1.6% with LBC. However, there was no statistically noteworthy difference in the disease detection rate among these two techniques [13]. This study also concluded that although the rate of unsatisfactory smears was reduced in LBC, as the detection rate of epithelial abnormalities is similar using both conventional Pap smear and LBC techniques, thus conventional Pap smear is still the better cervical screening method in the Indian scenario considering its cost‑effectiveness over LBC.

Taylor et al. studied the performance and accuracy of conventional Pap smear and LBC which were found to be statistically similar [37]. In another study, no statistically significant difference in sensitivity and specificity between the two different methods was reported for detection of CIN2 [38].

Studies by Sharma et al. [39] and Davey et al. [40] also showed that there was no discernible difference between LBC and CPS in the percentage of insufficient or poor smears.

A systematic review stated that conventional Pap and LBC have an equivalent performance in detection of squamous intraepithelial lesions and LBC is not more accurate. However, considering other clear advantages, including the possibility of aliquoting for the high-risk HPV test, LBC is gradually replacing conventional Pap smears [40].

The Pap test in its original preparation, which has been in use for more than 50 years also referred to as the “conventional cervical smear (CS) method for cytology collection”, is accepted even now for screening purposes thus remaining as a means for cervical cancer screening owing to its low cost and simplicity [17].

Karimi-Zarchi et al. conducted a study to compare conventional Pap smear, LBC, and colposcopy on a Pap smear containing atypical squamous cells [41]. This study revealed that compared to the two types of cervical cytology, colposcopy has a higher sensitivity in diagnosis of any cervical lesions. However, no statistically significant difference was observed in terms of specificity, sensitivity, positive predictive value, and negative predictive in the isolated comparison of the conventional Pap smear and LBC smear.

Patel et al. conducted a cross-sectional study to compare LBC and conventional Pap on cervical smears [15]. Conventional Pap smear exhibited a higher unsatisfactory rate (5%) as compared to LBC (1.4%). However, no substantial difference was noted between the two techniques in terms of the nonneoplastic reactive changes. Also, this study highlighted the importance of organisms, including pathogenic species and commensals. This is similar to the studies by Gupta et al. [42] and Singh et al. [43].

A prospective study was conducted to compare the usefulness of manual LBC with that of conventional Pap smear in cervical cancer screening. The low-cost manual LBC outlived conventional Pap smear in terms of diagnosis of cervical precursor lesions. It exhibited better cell morphology with amplified recognition of abnormalities. Additionally, there is a possibility of preservation of samples for cell block and ancillary studies like HPV detection and immunocytochemistry. Hence, it can be utilised for cervical cancer screening in limited resource settings as an alternate technique [14].

Sherwani et al. undertook a study to compare the sensitivity and specificity of LBC with that of the conventional Pap smear [44]. The study obtained the result 97.6 % and 53.7% sensitivity in LBC and conventional Pap smear respectively. However, the specificity was 50% in both the methods thus advocating LBC as a measure to improve early detection of cervical lesions.

The LBC method was superior to detecting squamous intraepithelial lesions especially HSIL when compared to the conventional Pap smear (1% versus 0.5 %) [45]. However, detecting the ASCUS (atypical squamous cells of unknown significance) and LSIL lesions was lower in LBC than the conventional Pap smear; also LBC demonstrated no significant change in detecting the lesions of squamous cell carcinoma and adenocarcinoma when compared to the conventional pap smear method.

Singh et al. [43] conducted a prospective study to compare the diagnostic efficacy of conventional Pap smear and LBC using “split samples” [43]. Although this study concluded that the rate of detection for cervical epithelial cell abnormalities was similar in both conventional Pap smear and LBC, it also stated that the LBC method accounts for a considerable reduction in the rate of unsatisfactory sample rate by offering better clarity (4.3% smears were reported as unsatisfactory by conventional method and 1.7% smears unsatisfactory by LBC technique), uniform spread of smears, less obscuring materials like hemorrhagic and inflammatory cells.

Kirschner et al. carried out a retrospective study to compare the diagnostic accuracy of conventional Pap smear and SurePath LBC in a population screening programme [46]. This study projected that the number of unsatisfactory samples was considerably reduced with LBC. The data put forward that although the detection rate of cervical precancerous lesions with LBC is increased, the number of false positive tests is considerably high. The specificity of the two tests however was statistically similar.

The comparison of diagnostic accuracy between LBC and conventional Pap smear continues to be a matter of great debate. While several studies have shown increased sensitivity of LBC over conventional Pap smear [47-50], others showed decreased or equal sensitivity and specificity [38,51,52].

## Conclusions

While the debate between the diagnostic efficacy of conventional Pap smear and LBC continues, there is no denying the fact that conventional Pap smear is convenient and easy to carry out and does not require high-end infrastructure unlike LBC. The expense of testing with these LBC techniques, which need a pricey automated device, limits their usefulness in underprivileged and developed countries. LBC offers an edge over conventional Pap smear as it provides a clearer microscopic picture and material can be retained for further testing. In the Indian scenario with a backdrop of being under-resourced, there is a necessity to re-consider the cost-effectiveness of the LBC technique as compared to conventional Pap smear, especially in the absence of HPV testing in bulk of the centres.

After Pap smear was initiated more than six decades ago, there have been a plethora of technological developments in the field of cervical cancer screening. The LBC method represents a weighty modification in the cervical cytology testing procedure. In India, although a shift from conventional Pap smear to LBC has been implemented in many centres, the conventional Pap smear still holds its importance as the method of choice in low-resource and field settings.

It can be concluded that as new technologies continue to develop, efforts must be cautious enough to warrant that the unmatched past success is extended to the future. As helpful as conventional Pap smears are in carrying out, LBC theoretically has few advantages over it. Most of the developing nations have adopted LBC as their first-line cervical screening method. Now if LBC is advantageous enough to completely replace conventional Pap smear in our under-resourced and underdeveloped Indian scenario is still an open question which will need further evaluation and investigations.
